# Association between Oxygen Consumption and Surface Electromyographic Amplitude and Its Variation within Individual Calf Muscles during Walking at Various Speeds

**DOI:** 10.3390/s21051748

**Published:** 2021-03-03

**Authors:** Kohei Watanabe, Shideh Narouei

**Affiliations:** Laboratory of Neuromuscular Biomechanics, Faculty of Liberal Arts and Sciences, School of International Liberal Studies, Chukyo University, Nagoya 466-8666, Japan; shnarouei@lets.chukyo-u.ac.jp

**Keywords:** walking, high-density surface electromyography, triceps surae, oxygen consumption, gastrocnemius

## Abstract

Surface electromyography (EMG) has been used to estimate muscle work and physiological burden of the whole body during human movements. However, there are spatial variations in surface EMG responses within individual muscles. The aim of this study was to investigate the relation between oxygen consumption and surface EMG responses of lower leg muscles during walking at various speeds and to quantify its spatial variation within an individual muscle. Nine young males walked on a treadmill at four speeds: preferred minus 1 km/h, preferred, preferred plus 1 km/h, and preferred plus 2 km/h, and the metabolic response was measured based on the expired gas. High-density surface EMG of the tibialis anterior (TA), medial gastrocnemius (MG), lateral gastrocnemius, and soleus muscles was performed using 64 two-dimensional electrode grids. Correlation coefficients between oxygen consumption and the surface EMG amplitude were calculated across the gait speeds for each channel in the electrode grid and for individual muscles. Mean correlation coefficients across electrodes were 0.69–0.87 for the four individual muscles, and the spatial variation of correlation between the surface EMG amplitude and oxygen consumption within an electrode grid was significantly greater in MG muscle than in TA muscle (Quartile deviations: 0.24 for MG and 0.02 for TA, *p* < 0.05). These results suggest that the physiological burden of the whole body during gait at various speeds can be estimated from the surface EMG amplitude of calf muscles, but we need to note its spatial distribution within the MG muscle.

## 1. Introduction

Walking is the most readily accessible form of exercise, but efficacy against age-related muscle dysfunctions or metabolic diseases such as type 2 diabetes mellitus may be limited if the walking speed is slow or comfortable [1,2]. Exercise intensity is a major component to identify physiological responses, and it is directly associated with the benefits of exercise in terms of working muscles and the whole body. Therefore, monitoring devices to estimate exercise intensity and related variables, i.e., heart rate, have been developed and are widely available commercially [3,4]. Exercise intensity is determined by contraction intensity at the working muscles. Therefore, physiological responses such as energy metabolism are determined by the “local” activity of working muscles. For example, walking at a vigorous intensity has been recommended to enhance glucose metabolism for type 2 diabetes mellitus patients [1]. This exercise-induced benefit strongly depends upon the contraction intensity of “local” working muscles. Therefore, the “local” activity of working muscles may be a more direct indicator and be useful to estimate exercise-induced physiological responses and benefits.

During walking, the calf muscles are the main contributors to propulsive force. The relative contribution of ankle plantar flexion torque to total positive work during walking is 73% in young adults [5]. Oi et al. (2003) reported that the soleus (SL) muscle showed the highest glucose uptake of all measured lower-extremity muscles during walking [6]. McGibbon (2003) reviewed age-related changes in gait pattern and suggested that age-related impairment in the ankle plantar flexion capacity limits the gait speed and step length [7]. Furthermore, it is well known that the role of the calf muscle in propulsion is related to the walking speed [8,9,10]. Therefore, monitoring the contraction levels of calf muscles would yield useful information to estimate physiological burden during walking and promote the benefits of walking as a form of exercise.

Surface electromyography (EMG) has been used to monitor and/or estimate the muscle contraction level or “local exercise intensity” during various human movements, including walking [11,12,13]. The surface EMG amplitude of lower-extremity muscles, including the calf muscles, increases with elevated physiological responses of the whole body, such as oxygen consumption, following an increase in walking speed [14,15,16]. However, simulation [17,18] and experimental [19,20,21,22] studies suggest that electrode location has a critical effect on surface EMG variables [23]. Taking into account this issue, spatial variation in surface EMG responses should be considered when surface EMG variables are used to estimate exercise intensity for both local muscles and the whole body. Because it would be difficult to control electrode location strictly among participants and users for the actual applications of monitoring devices with wearable electrodes [3,4,24] in various fields or clinical scenes, the effect of spatial variation in electrode location should be considered to record/monitor EMG and to estimate the physiological burdens from the recorded EMG. Future researchers/manufacturers may choose muscles with a higher correlation between EMG and the variable of whole-body physiological burden, i.e., oxygen consumption, and lower spatial variation of correlation between EMG and oxygen consumption such as lateral gastrocnemius (LG), SL, or tibialis anterior (TA). Alternatively, they may interpret inter-individual differences or variabilities in EMG-based estimation of oxygen consumption from the MG muscle as unavoidable intrinsic issues. Therefore, quantification of the relation between physiological burden such as oxygen consumption and recorded EMG and its spatial variation could help to determine the target to record surface EMG and interpret the results of surface EMG-based estimation of physiological burdens.

The aim of this study was to investigate the relation between oxygen consumption and surface EMG responses of lower leg muscles during walking at various speeds and its spatial variation within an individual muscle. This study would provide information to quantify and compare the strength of EMG as an indicator of physiological burden during walking among the muscles and its electrode location-dependent variations. Surface EMG was recorded from calf muscles including the medial gastrocnemius (MG), lateral gastrocnemius (LG), and SL muscles as plantar flexors and the tibialis anterior (TA) muscle as a dorsi flexor. We hypothesized that a strong correlation between surface EMG responses and oxygen consumption would be shown in MG, LG, and SL muscles but not in the TA muscle because of their contributions to propulsive functions during walking [15]. The arrangement between electrode pairs on the skin surface and muscle fibers/fascicles is one of the key determinants of surface EMG signal [17,25]. Based on anatomical properties, this factor is non-uniform along the muscle in the MG and LG muscles [23,26], but not in the SL and TA muscles. We, thus, also hypothesized that greater spatial variation would exist regarding the correlation between surface EMG responses and oxygen consumption in the MG and LG muscles than those in SL and TA muscles.

## 2. Methods

### 2.1. Participants

Nine young males participated in this study (age: 23.2 ± 5.2 years, height: 171.7 ± 7.0 cm, body mass: 61.7 ± 11.6 kg). They gave informed consent for the study after receiving a detailed explanation of the purposes, potential benefits, and risks associated with participation. All participants were healthy, with no history of any musculoskeletal or neurological disorders. All procedures used in this study were conducted in accordance with the Declaration of Helsinki and approved by the Research Ethics Committee for Human Experimentation at Chukyo University (No. 2019-058).

### 2.2. Experimental Design

The participants walked on a treadmill at four different walking speeds: preferred minus 1 km/h, preferred, preferred plus 1 km/h, and preferred plus 2 km/h, for 2 min each. The order of the different walking speeds was randomized. The preferred walking speed was determined from three trials involving a 10-m walk on a flat floor before the treadmill trials. During walking on the treadmill, surface EMG and oxygen consumption ($\dot{V}$O_2_) were measured.

Expired gas was sampled through a mask covering the mouth and nose during walking using the breath-by-breath method (AE310S, Minato Medical Science Co., Ltd., Osaka, Japan) [27]. From the sampled gas, $\dot{V}$O_2_ was calculated and averaged for 10 gait cycles, over which the surface EMG was analyzed during the final 30 s of 2 min of walking.

### 2.3. Surface EMG Recording

**Figure 1:**
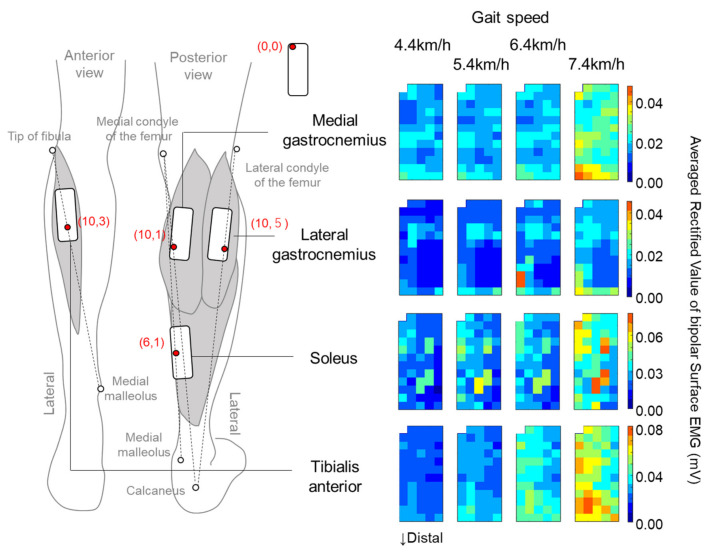
Electrode locations for individual muscles and representative data of averaged rectified values of bipolar surface electromyography (EMG) shown by a color map at four different walking speeds. Red circles in the left panel indicate the electrode locations recommended by SENIAM (Surface ElectroMyoGraphy for the Non-Invasive Assessment of Muscles).

Multichannel surface EMG signals were recorded from the MG, LG, SL, and TA muscles of the left leg during the treadmill walking. We used a semi-disposable adhesive grid of 64 electrodes with a 1-mm diameter and 8-mm interelectrode distance (GR08MM1305, OT Bioelettronica, Torino, Italy). The electrodes were organized in 13 rows and 5 columns of electrodes with one electrode missing in the upper left corner. The covered area for an electrode grid is approximately 33 × 97 mm. The locations of the electrode grid for individual muscles were determined as follows: (1) all electrodes of the grid were located on the muscle of interest; (2) one of the electrodes corresponded to the electrode locations recommended by the project of Surface ElectroMyoGraphy for the Non-Invasive Assessment of Muscles (SENIAM) [28]; (3) the same row and column of the matrix were used for the electrode recommended by SENIAM among the participants, and (4) long sides of electrode grids were arranged along the estimated fiber directions [28] (Figure 1).

The superficial area of the MG, LG, SL, and TA muscles was identified by ultrasonography (LOGIQ e Premium, GE Healthcare), and the electrode grids were located within the identified area. In each grid, one of the electrode channels corresponded to the electrode location recommended by SENIAM as follows: MG, proximal 33% of the line between medial epicondyle of femur and calcaneus; LG, proximal 33% of the line between lateral epicondyle of femur and calcaneus; SL: 66% of the line between medial epicondyle of femur and medial malleolus; TA, proximal 33% of the line between tip of the fibula and medial malleolus (Figure 1). Monopolar surface EMG signals were recorded and amplified by a factor of 500, sampled at 2048 Hz, and converted to digital form by a 16-bit analog-to-digital converter (Quattrocento, OT Bioelettronica, Torino, Italy). Recorded monopolar surface EMG signals were transferred to analysis software (MATLAB R2018a, MathWorks GK, Tokyo, Japan), filtered by a band-pass filter (10–450 Hz), and differentiated between neighboring electrodes along the columns. This resulted in 59 channels of bipolar surface EMG for each muscle. For each channel, the averaged rectified value (ARV) of the signal was calculated over the 10 gait cycles during the final 30 s of 2 min of walking at each of the four different walking speeds (Figure 1).

### 2.4. Data Analysis and Statistics

**Figure 2:**
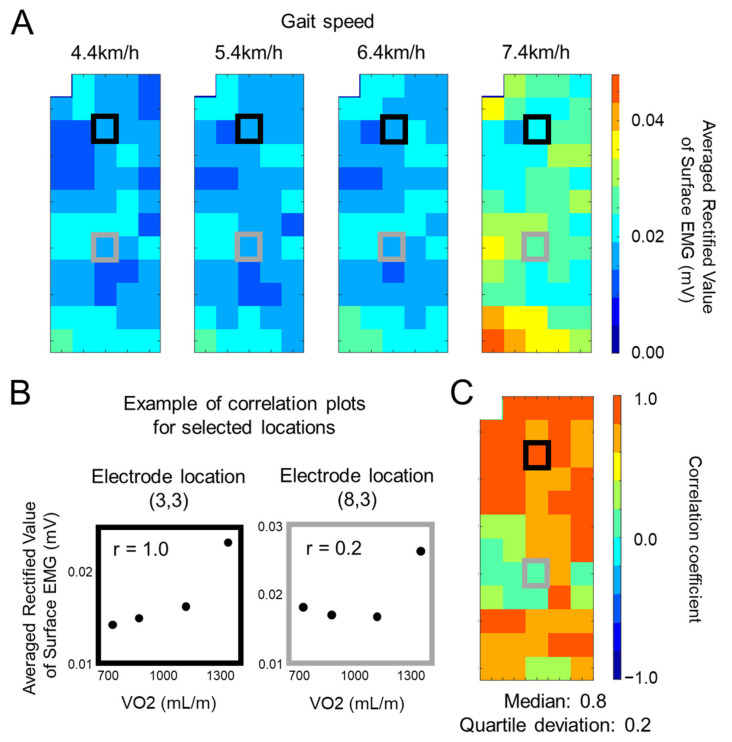
Representative data from the medial gastrocnemius (MG) muscle of a single participant. (**A**) Averaged rectified value (ARV) of surface electromyography at each of four different walking speeds. (**B**) Examples of correlation analysis between oxygen consumption (VO2) and averaged rectified value of surface electromyography at each of two selected electrode locations. Black and gray panels correspond to the electrode locations with black and gray boxes in (**A**) and (**C**). (**C**) Spatial distribution of correlation coefficients across all recorded channels.

Correlation coefficient values between oxygen consumption and the ARV of surface EMG for each electrode location and for individual muscles across the four different walking speeds were calculated using Spearman’s rank correlation coefficient (Figure 2A,B). Results of correlation coefficient analysis were plotted on a matrix (Figure 2C) and their median and quartile deviation values across the channels were calculated for quantification of the level of correlation and spatial variations within a muscle.

Non-parametric tests were used in this study since the sample size was small (*n* = 9) and the Shapiro–Wilk test showed that the distribution of data was partly non-Gaussian. Friedman’s tests were performed to examine the effect of the muscle on the median and quartile deviation values of correlation coefficients between oxygen consumption and the ARV of surface EMG. When significant effects of the muscle were detected by Friedman’s test, Dunn’s test with the Bonferroni correction was performed as a post-hoc test for the pairs between muscles.

The level of significance was set at 0.05 and statistical analyses were performed using MATLAB (version R2015b, Math Works GK, Tokyo, Japan) and SPSS software (version 21.0; SPSS, Tokyo, Japan). For Dunn’s test, used as a post-hoc test, the level of significance was modified by the Bonferroni correction, i.e., 0.05/number of compared pairs (0.05/6 = 0.0083). The effect size for the Friedman test and its post-hoc test were calculated as the Kendall’s *W* test value (*W*) and correlation coefficient (*r*), respectively [29,30].

## 3. Results

The mean preferred walking speed was 5.16 ± 0.60 km/h. Thus, walking speeds of preferred minus 1 km/h, preferred plus 1 km/h, and preferred plus 2 km/h were 4.16 ± 0.60, 6.16 ± 0.60, and 7.16 ± 0.60 km/h, respectively.

Mean oxygen consumptions at preferred minus 1 km/h, preferred, preferred plus 1 km/h, and preferred plus 2 km/h were 850.1 ± 198.4, 1032.1 ± 226.1, 1266.9 ± 296.7, and 1602.5 ± 339.6 mL/min, respectively.

**Figure 3:**
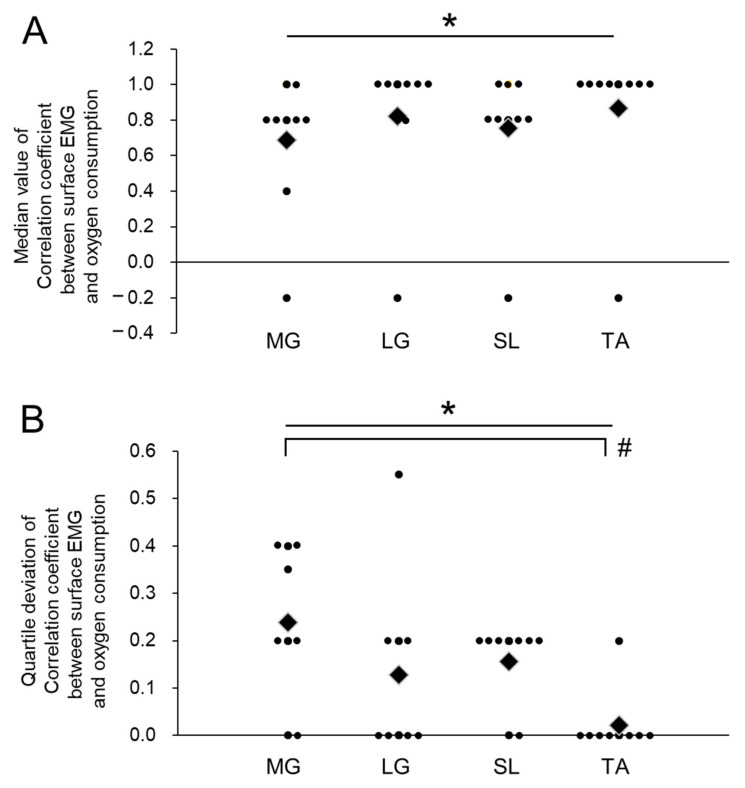
Median values (**A**) and quartile deviation (**B**) of correlation coefficients in electrode grids for individual muscles. Diamonds and circles indicate the mean values and individual participants, respectively. * *p* < 0.05, significant effect of muscle, # *p* < 0.0083, significant difference between muscles.

Median correlation coefficients between the ARV of surface EMG and oxygen consumption were 0.69 ± 0.38 for the MG muscle, 0.82 ± 0.39 for the LG muscle, 0.76 ± 0.37 for the SL muscle, and 0.87 ± 0.40 for the TA muscle (Figure 3A). There were significant effects of the muscle on median correlation coefficients between oxygen consumption and the ARV of surface EMG (*p* < 0.05, *W* = 0.57), but there were no significant differences between the muscles in post-hoc tests (Figure 3).

Quartile deviations of correlation coefficients between the ARV of surface EMG and oxygen consumption were 0.24 ± 0.16 for the MG muscle, 0.13 ± 0.19 for the LG muscle, 0.16 ± 0.09 for the SL muscle, and 0.02 ± 0.07 for the TA muscle (Figure 3B). Significant effects of the muscle were detected in quartile deviations of correlation coefficients between oxygen consumption and the ARV of surface EMG (*p* < 0.05, W = 0.78), and a significant difference between MG and TA muscles was observed in post-hoc tests (*p* < 0.0083, *r* = 0.80) (Figure 3B).

## 4. Discussion

We tested the relation between oxygen consumption and surface EMG responses of lower leg muscles during walking at various speeds and its spatial variation within an individual muscle. The results showed high correlation coefficients between the ARV of surface EMG and oxygen consumption for all recorded lower leg muscles: the MG, LG, SL, and TA muscles. This result failed to support our first hypothesis that high correlations between surface EMG responses and oxygen consumption are shown only in the calf muscles. Greater spatial variations in correlation coefficients between the ARV of surface EMG and oxygen consumption were noted in the MG muscle, as we had hypothesized. However, spatial variation of the LG muscle was not greater than other muscles, meaning that factors except for arrangement between electrode pairs on the skin surface and muscle fibers/fascicles may contribute to spatial variations in the correlation coefficients, such as regional neuromuscular regulation [19,20,31].

The present study showed a proportional increase in oxygen consumption with the increase in walking speed. This means that metabolic responses and the whole-body physical burden were increased with the four different walking speeds adopted in the present study. We also confirmed that the ARV of surface EMG linearly rose with increasing walking speed at most channels of the recorded muscles (e.g., Figure 2A,B). As a result, high correlation coefficients between the ARV of surface EMG and oxygen consumption were noted in the four muscles (0.69–0.87) (Figure 3A). Calf muscles such as the MG, LG, and SL muscles produce ankle plantar flexion joint torque and have been considered major contributors to propulsion on walking [8,9,10]. Previous studies reported increases in ankle plantar flexor joint power and surface EMG amplitudes in the MG and SL muscles with an increase in walking speed [32,33] and the dominant contribution of ankle plantar flexors to create output over the gait cycle [10,15,34]. Therefore, high positive correlations between the ARV of surface EMG recorded from the calf muscles and oxygen consumption observed in the present study may be reasonable. On the other hand, we also noted a high correlation between the ARV of surface EMG and oxygen consumption in the TA muscle. Since ankle dorsi flexion joint momentum is too low and does not contribute to propulsion during walking [8,15], we hypothesized that the correlation between the ARV of surface EMG and oxygen consumption could not be detected in the TA muscle, and so the TA muscle was also tested as a control or minor contributory muscle in the present study. The TA muscle is activated mainly during the swing phase to the beginning of the stance phase where the propulsive force is not produced in the leg on the side of interest [11,13]. However, increases in surface EMG amplitudes in the TA muscle with an elevated walking speed were observed during the swing phase to beginning of the stance phase [32]. These activations could be associated with successful heel contact and toe clearance during movements from swing to stance phases. With an increase in walking speed, the maximum ankle plantar flexion angle after toe-off and during the mid-swing phase increases (van Hedel et al., GP 2006). We, thus, considered that greater activation may be required for dorsi flexion muscles such as the TA muscle following an increase in walking speed in order to return to the necessary ankle joint angle during swing movements [35] to achieve successful toe clearance.

As we hypothesized, greater spatial variations in the coefficient correlation between the ARV of surface EMG and oxygen consumption were noted in the MG muscle (Figure 3B). For this muscle, region-specific EMG responses were reported along its longitudinal axis. Previous studies showed a regional distribution in manifestations of fatigue and selective activation of the distal region during standing within the MG muscle [19,21]. These phenomena can be explained by localized recruitments of muscle fibers along the MG muscle that can be confirmed by multi-channel surface EMG and intramuscular EMG techniques [20]. On the other hand, relatively lower spatial variations in the coefficient correlation between the ARV of surface EMG and oxygen consumption were noted in the LG, SL, and TA muscles (Figure 3B). We set the electrode grid in areas where we can actually attach an electrode pair, such as the muscle belly, and the electrode grid covered the electrode location recommended by SENIAM [28]. Since these muscles showed higher correlation coefficients between the ARV of surface EMG and oxygen consumption with lower spatial variation, the physiological burden on the whole body could then be estimated from surface EMG responses with higher validations without the need to pay attention to the electrode location when electrodes were attached around the muscle belly for the LG, SL, and TA muscles.

In the present study, the correlation with oxygen consumption was calculated for the ARV of surface EMG sampled across ten whole gait cycles, and we did not consider the gait cycle phases. Greater positive correlations would be observed if we sampled the phases where the muscles of interest activate to elicit joint movements that directly contribute to an increase in walking speed. For example, activations of the SL muscle would be highly correlated with an increase in the walking speed during the stance phase when ankle plantar flexion joint torque directly translates to the ground, but not during the swing phase when generated joint torque is not transferred to the ground. However, it is difficult to separate oxygen consumption and whole-body physiological responses into individual gait phases. Moreover, the recording of surface EMG or other biomedical signals with high time resolution and its separation into given epochs would require an additional calculation process and extra cost when applying this concept to commercially developed products. We thus calculated the ARV across the gait cycle to estimate the relation with oxygen consumption during walking. Future studies are needed to further validate the correlation between surface EMG and whole-body physiological responses with gait phase-based analysis.

Based on the results of the present study, future researchers/manufacturers that aim to estimate physiological burden during walking from EMG of lower leg muscles may choose the muscles with higher correlations between EMG and oxygen consumption with lower spatial variation, such as LG, SL, or TA. Furthermore, they may interpret inter-individual differences or variabilities in EMG-based estimations of oxygen consumption in the MG muscle as unavoidable intrinsic issues.

In conclusion, the present study investigated the relation between oxygen consumption and the surface EMG amplitude and spatial variation of the relation between oxygen consumption and the surface EMG amplitude within individual lower leg muscles during walking at various speeds. We noted strong positive correlations between oxygen consumption and the surface EMG amplitude across the various walking speeds in the MG, LG, SL, and TA muscles. While the LG, SL, and TA muscles showed relatively low spatial variations across the multiple electrode locations in correlation coefficients with oxygen consumption, greater spatial variations in correlation coefficients between oxygen consumption and surface EMG amplitude were observed within the MG muscle. These results suggest that the surface EMG amplitude recorded from around the muscle bellies of lower leg muscles can be used to estimate the physiological burden on the whole body during walking at various speeds, but the electrode location should be carefully considered for the MG muscle.

## Data Availability

Data are available from the authors upon reasonable request.

## Abbreviations

ARV	Averaged rectified value
EMG	Electromyography
LG	Lateral gastrocnemius
MG	Medial gastrocnemius
SL	Soleus
TA	Tibialis anterior
$\dot{V}$O_2_	Oxygen consumption
